# *RRM1* *151A>T, *RRM1* ‐756T>C, and *RRM1* ‐585T>Gis associated with increased susceptibility of lung cancer in Chinese patients

**DOI:** 10.1002/cam4.703

**Published:** 2016-06-23

**Authors:** Xiao‐Ling Xu, Ji Zheng, Wei‐Min Mao, Zhi‐Qiang Ling

**Affiliations:** ^1^ Zhejiang Cancer Research InstituteZhejiang Province Cancer Hospital, Zhejiang Cancer CenterNo.38 Guangji Rd.Banshanqiao DistrictHangzhou310022China; ^2^ Key laboratory on Diagnosis and Treatment Technology on Thoracic CancerHangzhouZhejiang310022China; ^3^ Department of Thoracic Tumor SurgeryZhejiang Province Cancer Hospital, Zhejiang Cancer CenterNo.38 Guangji Rd.Banshanqiao DistrictHangzhou310022China

**Keywords:** DNA repair, lung cancer, polymorphisms, ribonucleotide reductase M1, SNP

## Abstract

Ribonucleotide reductase M1 (*RRM1*) is a crucial gene in DNA repair. Recent studies have shown that *RRM1* expression can be a powerful predictor of survival or chemotherapy sensitivity in patients presenting with carcinomas who are treated with adjuvant gemcitabine‐based chemotherapy including lung cancer. However, the relationship between the single nucleotide polymorphisms (SNP) of *RRM1* and the susceptibility of lung cancer to chemotherapy has not been well addressed. We detected six tag SNPs of *RRM1* genotypes in a cohort of 1007 patients with primary lung cancer and 1007 age‐ and sex‐matched population controls using SNaPshot detection technology. Logistic regression, odds ratios (OR), and 95% confidence intervals were calculated to estimate lung cancer risk associated with SNP genotypes and haplotypes, after adjusting for case–control matching factors. Compared with the T/T and A/T genotype of *RRM1* *151A>T, the A/A genotype had an increased risk for overall lung cancer (adjusted OR, 1.33). Additionally, the T/T+T/C genotypes of *RRM1* ‐756T>C were risk factors that increased the susceptibility to lung cancer (adjusted OR 1.54, as compared with the C/C genotype). While the T/T+G/T genotypes of *RRM1* ‐585T>G behaved as protective factors to increase the susceptibility to lung cancer (adjusted OR 0.44, as compared with the C/C genotype). In summary, this is the first study to systematically identify the relationship between the polymorphisms of *RRM1* and individual susceptibility to lung cancer. It is anticipated that the *RRM1* *151A>T, *RRM1* ‐756T>C, and *RRM1* ‐585T>G polymorphisms will improve the predictive prognosis of lung cancer sensitivity.

## Introduction

Lung cancer is the most frequent solid tumor and the leading cause of cancer‐related deaths in both developing and developed countries, which is a major public health problem worldwide 1, 2. Early stage (I/II) non–small‐cell lung cancer (NSCLC) and small‐cell lung cancer (SCLC) are considered to have a better 5‐year survival rates (45% and 31%, respectively) than that at advanced stage (III/IV) 3. Thus, to identify certain population who may have susceptibility to lung cancer and diagnose lung cancer in early stage are crucial to improve treatment outcome. Since the genetic characteristics has been proved to contribute to lung cancer development, many molecular epidemiological studies have been conducted to evaluate the relationship between lung cancers and the genetic variety, such as single nucleotide polymorphisms (SNP) in genes which may be involved in lung cancer development.

The DNA repair system plays an important role in protecting against mutagenesis and carcinogenesis. These genes are mainly involved in DNA maintenance and repair, carcinogen metabolism, cell cycle regulation, apoptosis, and so on. Some of the SNPs have been consistently shown to correlate with lung cancer susceptibility, including XPD 4, APEX, XRCC 5, POLG2, RECQL4 6, and so on.

The ribonucleotide reductase M1 (*RRM1*) gene, a crucial gene of the nucleotide excision repair (NER) system, is important in the DNA damage removal pathway, which encodes the regulatory subunit of ribonucleotide reductase. This is the rate‐limiting step in deoxyribonucleotide formation, and the only known enzyme that converts ribonucleotides to deoxyribonucletides, which is required for DNA polymerization and repair. Furthermore, *RRM1* is the molecular target of gemcitabine, and its mRNA expression levels are related to the efficacy of gemcitabine therapy 7, 8. The *RRM1* polymorphism – 37 C<A and haplotype – was shown to be associated with susceptibility to gemcitabine in cancer patients including lung cancer 9, 10. However, the relationship between the ribonucleotide reductase M1 (*RRM1*) gene and susceptibility to lung cancer has not been well addressed.

## Material and Methods

### Study subjects

Patients, diagnosed with lung cancer based on the pathological features, were recruited between June 2009 and August 2012 at Zhejiang Cancer Hospital. After omitting samples that failed to be genotyped, the final number of study subjects consisted of 1007 patients with primary lung cancer and 1007 age‐ and sex‐matched population controls. In cancer cases, the peripheral blood samples were collected from patients with primary lung cancer who had not previously received surgical treatment or radiotherapy or chemotherapy.

### SNP target determinations

For SNP site selection, we searched potential functional polymorphisms in SNP in the 5′ promoter region, nonsynonymous SNP, SNP in the 5′ untranslated region, synonymous SNP in the 3′ untranslated region, and synonymous SNP. In addition, tagSNPs were identified to examine common patterns of variation in the *RRM1* gene regions. All SNPs were selected to account for all having a minor allele frequency (MAF) of 5% from the HapMap database. Haplotype frequencies and the linkage disequilibrium coefficient were estimated using Haploview software 4.2 11. HapMaptag SNPs in a pairwise linkage with a disequilibrium threshold were defined by a correlation coefficient (*R* ^2^) of 0.80. Hardy–Weinberg proportions in the controls and haplotypes were assessed by Fisher's exact test.

### Multiplex polymerase chain reaction

Genomic DNAs were extracted from peripheral blood mononuclear cells using the blood genomic DNA isolation kit (Axygen Scientific Inc., 33210 Central Avenue, Union City, California 94587, CA, USA). The reaction system of PCR was as follows: 20 *μ*L of 1X GC‐I buffer (Takara Bio Inc., Otsu, Shiga, Japan), 3.0 mmol/L Mg^2+^, 0.3 mmol/L dNTP, 1 U HotStar Taq polymerase (Qiagen Co., Ltd., Duesseldorf, Germany), 1 *μ*L DNA sample, and 2 *μ*L multiplex PCR primers. The PCR cycle was designed as following: predenaturing at 94°C for 2 min, denaturing at 94°C for 20 sec, annealing at 65°C for 20 sec, extension at 72°C for 30 sec, total 11 cycles; then 94°C for 20 sec, 59°C for 30 sec, 72°C for 90 sec, total 24 cycles; and extension at 72°C for 2 min.

### SNaPshot multiplex single base extension reaction

Extension reaction (10 *μ*L) included 5 *μ*L buffer from the SNaPshot Multiplex Kit (Applied Biosystems Co., Ltd., Foster City, CA, USA), 2 *μ*L multiple PCR products after purification, 1 *μ*L extension primer mix, and 2 *μ*L of ultrapure water. The conditions of the PCR amplification were as follows: 96°C for 1 min followed by 96°C for 10 sec, 55°C for 5 sec, 60°C for 30 sec, which was repeated for 28 cycles, and then held at 4°C. Next, 0.5 *μ*L of a Liz120 size standard and 9 *μ*L of a Hi‐Di mix were placed into 0.5 *μ*L of the purified extension product, which was then denatured at 95°C for 5 min, then sequenced using an ABI 3730XL DNA analyzer. The original data were analyzed by GeneMapper 4.1 software (Applied Biosystems Co., Ltd., Foster City, California, USA).

### Statistical analysis

Differences in gender, age, and genotypic frequencies that were found between cases and control subjects were evaluated by Pearson's chi‐square (χ^2^) test. The dominant and recessive models were used to assess the risk of the SNP genotype in lung cancer. The reference group was the minor homozygous genotype among controls. The associations between *RRM1* genotypes and risk of lung cancer were estimated by computing the odds ratios (ORs) and their 95% confidence intervals (CIs) from both univariate and multivariate logistic regression analyses. The ORs were also adjusted for age and gender by the use of frequency matching. These statistical analyses were performed with the SPSS statistical software package (SPSS v.18.0; Chicago, IL, USA). All probability values were two sided and *P* values with an alpha value <0.05 were considered statistically significant.

## Results

### Patient characteristics

**Figure 1:**
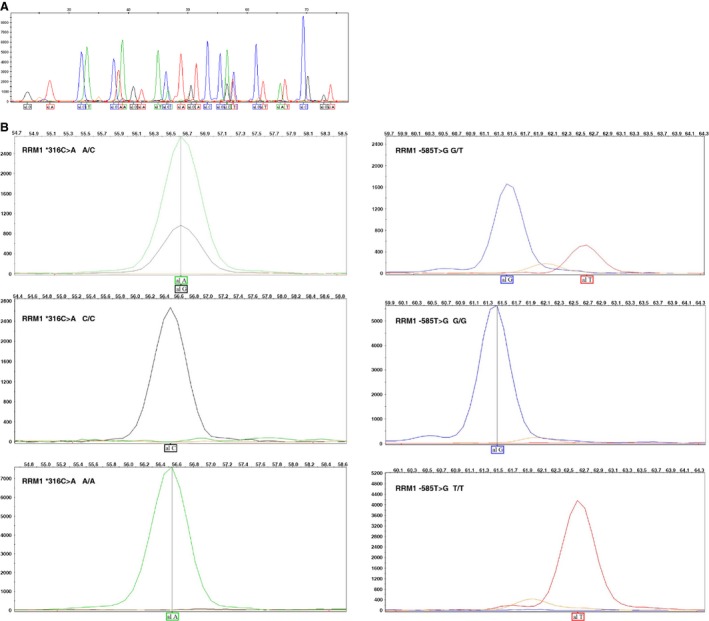
Typical genotype plots of SNaPshot detection technology. The plot for genotyping of (A) multiple SNPs for one sample (control no. 499) and (B) *RRM1* ‐ 316C>A and *RRM1* ‐ 585T>G.

For SNP genotyping, more than 99% of the samples were successfully determined and a representative plot for genotyping is shown in Figure 1A and B. There were no statistically significant differences between cases and controls in frequency distribution of sex, age, and lifestyle in the 1007 patients with lung cancer and 1007 controls (all *P*s > 0.05). Allelic distributions in controls were in good agreement with those in the CHBHapMap populations. All genotypic distributions were compatible with the Hardy–Weinberg equilibrium (*P *>* *0.05). Of the 1007 lung cancer cases, 277 (27.5%) were classified as squamous cell carcinoma (SCC), 405 (40.2%) as adenocarcinomas, 150 (14.9%) as small cell lung cancers (SCLC), and 175 (17.4%) as others carcinomas. These patients included undifferentiated cancers, bronchioalveolar carcinomas, and mixed cell carcinomas.

### Association between SNPs and risk of lung cancer

The *RRM1* genotypes identified by SNaPshot detecting technology were readily discerned. We have genotyped six tagSNPs in *RRM1* including *RRM1* *151A>T, *RRM1* *316C>A, *RRM1* ‐756T>C, *RRM1* ‐269C>A, *RRM1* ‐702G>A, and *RRM1* ‐585T>G. Among them, *RRM1* *151A>T and *RRM1* *316C>A were complete linkage (*R* ^2^ = 1). Figure 1 shows representative pictures for genotyping of the *RRM1* *316C>A and *RRM1* ‐585T>G.

Genotyping data frequencies for six tagSNPs in *RRM1* are shown in Table 1. The distributions of these *RRM1* genotypes were then compared, respectively, among cases and controls. It was found that the frequencies of *RRM1* *151A>T A/A, A/T, and T/T genotypes among cancer cases were significantly different as those among controls (6.8%, 43.3%, and 49.9% vs. 7.0%, 41.9%, and 51.1%, *P *=* *0.001). There were also significant differences observed in *RRM1* ‐702G>A and *RRM1* ‐585T>G (*P *=* *0.03 and *P *=* *0.01). However, the frequencies of *RRM1* ‐756T>C and *RRM1* ‐269C>A genotypes were almost the same as those among in cancer cases and controls (*P *=* *0.11 and *P *=* *0.19).

**Table 1:** *RRM1* genotypes of cases and controls and their association with risk of overall lung cancer

SNP	Function	Genotype	Patients (*n* = 1007)	Control subjects (*n* = 1007)	Dominant model	*P* value	Recessive model	*P* value
			*N* (%)	*N* (%)	Adjusted OR^b^ (95% CI)		Adjusted OR (95% CI)	
*RRM1* *151A>T^a^	3′ UTR	A/A	68 (6.8)	70 (7.0)	1.53 (0.96–2.45)	0.08	1.33 (1.11–1.60)	**0.00**
		A/T	436 (43.3)	364 (36.1)			Ref.	
		T/T	503 (49.9)	573 (56.9)	Ref.			
*RRM1* ‐756T>C	Promoter	T/T	70 (7.0)	96 (9.5)	1.54 (1.10–2.15)	**0.01**	0.94 (0.57–1.54)	0.79
		T/C	422 (41.9)	416 (41.3)			Ref.	
		C/C	515 (51.1)	508 (50.4)	Ref.			
*RRM1* ‐269C>A	5′ UTR	A/A	526 (52.2)	508 (50.4)	0.80 (0.35–2.27)	0.81	1.05 (0.64–1.73)	0.86
		C/A	411 (40.8)	416 (41.3)			Ref.	
		C/C	70 (7.0)	96 (9.5)	Ref.			
*RRM1* ‐702G>A	Promoter	A/A	31 (3.1)	35 (3.5)	1.18 (0.60–2.30)	0.64	1.04 (0.75–1.44)	0.83
		G/A	357 (35.5)	303 (30.1)			Ref.	
		G/G	619 (61.5)	669 (66.4)	Ref.			
*RRM1* ‐585T>G	3′ UTR	T/T	46 (4.6)	31 (3.1)	0.44 (0.24–0.84)	**0.01**	0.95 (0.54–1.67)	0.84
		G/T	445 (44.2)	396 (39.3)			Ref.	
		G/G	516 (51.2)	580 (57.6)	Ref.			

SNP: single nucleotide polymorphism; OR: odds ratio; CI: confidence interval; Ref: reference; UTR: untranslated region.

a *RRM1* *151A>T and *RRM1* *316C>A indicates complete linkage, we enrolled only one of them to the logistic analysis.

b ORs were adjusted for sex and age in a logistic regression model. There was significant difference, *P* < 0.05.

We used logistic regression analysis to examine potential associations between polymorphisms in the *RRM1* genes and the risk of lung cancer after adjusting for age, gender, and lifestyles. Adjusted ORs are represented in Table 1. Both dominant and recessive models were considered for each SNP.

Compared with the T/T and A/T genotypes of *RRM1* *151A>T, the A/A genotype had an increased risk for overall lung cancer (adjusted OR, 1.33; 95% CI, 1.11–1.60) by recessive models. Additionally, the T/T+T/C genotypes of *RRM1* ‐756T>C behaved as risk factor of enhanced susceptibility of lung cancer (adjusted OR 1.54; 95% CI, 1.10–2.15) as compared with the C/C genotype. While the T/T+G/T genotypes of *RRM1* ‐585T>G behaved as protective factors in increasing the susceptibility of lung cancer (adjusted OR, 0.44; 95% CI, 0.24–0.84) as compared with the C/C genotype. However, there was no evidence that showed any association between the heterozygous genotypes of both *RRM1* ‐269C>A and *RRM1* ‐702G>A sites and relative risk of lung cancer development.

### Association between SNPs and risk of lung cancer pathology subtypes

Since lung cancers are derived from different cell types and carcinogenesis of different subtypes of lung cancer may be initiated by diverse DNA damage insults, the risk related to the *RRM1* polymorphisms was further evaluated among non–small‐cell lung cancer (NSCLC) and SCLC.

There were 857 patients with NSCLC and 150 patients with SCLC among the total of 1007 lung cancer cases. In dominant models, it was found that *RRM1* *151A>T had more than eightfold increased risk associated with the polymorphisms in patients with SCLC, with an adjusted OR of 8.54 (95% CI, 1.02–71.3; *P *=* *0.48) for the A/A+A/T genotype as compared with the T/T genotype. However, there was no evidence that showed any association between the polymorphisms of *RRM1* and SCLC. In addition, we found that there was a significant association between the polymorphisms of *RRM1* ‐585T>G and NSCLC with an adjusted OR of 0.62 (95% CI, 0.36–1.05; *P *=* *0.07) by dominant models in patients with non–small‐cell lung cancer. Thus, the *RRM1* ‐756T>C had an adjusted OR of 1.72 (95% CI, 1.19–2.48; *P *=* *0.00). In the recessive model, *RRM1* *151A>T had a remarkable association with the susceptibility of lung cancer with 1.43 (95% CI, 1.17–1.74; *P *=* *0.00).

The potential interactions between these six genetic polymorphisms and age on risk of lung cancer were analyzed in a logistic regression model. However, no effect was observed (data not shown), apparently due to the small numbers involved.

### Haplotype analysis

The haplotype frequencies of *RRM1*among cases and controls were estimated and the results are presented in Table**** 2. No significant difference was observed between cases and controls in terms of haplotype frequencies of the *RRM1* *151A>T and *RRM1* *316C>A polymorphisms. We also performed linkage disequilibrium analysis to examine the linkage between the polymorphisms at the two loci. The two locus disequilibrium was statistically significant (*R* ^2^ = 1), which indicated that the two polymorphisms were completely linked in our study population. There was no significant linkage disequilibrium found among the other four polymorphisms of *RRM1* for R^2^ < 0.8.

**Table 2:** Two‐marker haplotype analysis

Haplotype(*RRM1* *151A>T and *316C>A)	Cases (%)	Controls (%)	OR (95% confidence interval)	*P* value
TA	721 (71.6)	755 (75.0)	1.09 (0.69–1.03)	0.09
AC	286 (28.4)	252 (25.0)		

OR: odds ratio; CI: confidence interval.

## Discussion

Carcinogenesis is a multistep process which involved genetic and environmental risk factors. Common polymorphisms in many genes, including those involved in DNA repair, have been shown to have individual susceptibility to cancers. It has been proved that polymorphisms of DNA repair genes, such as the polymorphisms of XPA ‐4G>A (rs1800975), ERCC2 862G>A (rs1799793) 12, MSH3 3133G>A (rs26279), and PMS1 639G>A (rs5742938) 13, are associated with the risk of lung cancer.

*RRM1* is involved in several important DNA repair processes (e.g., nucleotide excision repair, interstrand cross‐link repair, ROS‐induced single‐strand break repair, and double‐strand break repair), and it resides on chromosome segment 11p15.5 14 in a region of frequent loss of heterozygosity (LOH). Clinical and cell biological studies suggest that LOH is an important tumor‐suppressor gene region in lung cancer 15, 16. Moreover, *RRM1* is a metastasis suppressor gene through PTEN‐regulated pathways in lung cancer 17. Furthermore, it encodes the regulatory subunit of ribonucleotide reductase which was inhibited by gemcitabine metabolites (5′ diphosphate) 18. In a recent study, ribonucleotide reductase might play a key role in suppressing the invasive capacity and anchorage‐independent growth of human lung cancers through the ability of the ribonucleotide reductase small subunit p53R2 to bind ERK kinase 2, thereby suppressing MEK‐ERK activity 19.

Many recent works have mainly focused on whether *RRM1* can be used as a molecular predictor in lung cancer by different methods of biomarker analysis, including quantitative real‐time PCR analysis, immunohistochemistry, western blot analysis, and automated quantitative analysis 7, 20, 21, 22. A meta‐analysis of 18 studies evaluating a gemcitabine‐based regimen in patients with advanced NSCLC (*n* = 1243) was recently published 21. Patients with low tumor *RRM1* expression assessed using either qRT‐PCR or IHC survived 3.94 months longer (95% CI, 2.15–5.73; *P *=* *0.001) than those patients with high *RRM1* expression. Meanwhile, *RRM1* expression has been shown to be a powerful predictor of survival or chemotherapy susceptibility in patients with carcinomas, such as pancreatic cancer 23, advanced nasopharyngeal carcinoma 24, and gastric cancer 25, treated with adjuvant gemcitabine‐based chemotherapy. Since the specimen of core needle biopsies derived from patient tumors are always inconvenient to obtain, and the technologies of immunohistochemical methods, mRNA expression and quantitative in situ protein analyses, are complicated, it is necessary to identify a more generally applicable methodology. Genotyping of SNPs is more convenient and stable; additionally, genotyping is assisted by the relative ease of obtaining peripheral blood. However, whether the polymorphism of *RRM1* was related to individual risk of developing lung cancer has not been well investigated.

Genetic polymorphisms may affect protein structure, function, stability, or folding. The most common form of polymorphism in the human genome is an SNP, and some SNPs have been shown to correlate with cancer sensitivity including lung cancer. In this study, we selected six SNP sites in *RRM1*, four sites located at 3′ untranslated region (UTR), one located at 5′ UTR, and the other two sites were located at the promoter. In addition, microRNAs, 21–24 nucleotide‐long small noncoding RNA gene products, can regulate gene expression by base pairing with the 3′ UTR region of target mRNAs.

Finally, we identified three common polymorphisms of *RRM1* that were associated with lung cancer in the Chinese population. On the basis of the analysis of 1007 patients and 1007 frequency‐matched controls, the A/A genotype of polymorphism *RRM1* *151A>T and the T/T+T/C genotype of *RRM1* ‐756T>C was positively associated with an increased risk to the disease. While the T/T+G/T genotype of *RRM1* ‐585T>G was associated with a decreased risk for tumorigenesis, suggesting that these SNPs may have potential function to influence the amplification and expression of *RRM1*. Subgroup analysis was also conducted, and polymorphisms of *RRM1*were both associated with NSCLC and SCLC.

However, our study also has its limitations. The primary limitation of our study was the use of a single center study and consequently low power of analysis in statistical measurements, which can lead to both false negative as well as false positive findings. Moreover, such findings should thus be considered preliminary 26.

In summary, to the best of our knowledge, this is the first study to systematically identify the relationship between the tagSNPs of *RRM1* and individual susceptibility to lung cancer. Hopefully, the *RRM1* *151A>T, *RRM1* ‐756T>C, and *RRM1* ‐585T>G will improve the prediction of lung cancer sensitivity. However, these results suggest a few potential candidates for further investigation in larger studies. Furthermore, the regulatory pathway associated with potentially surrogate SNPs remains to be explored.

## Conflict of Interest

None declared.
